# Macrophage migration inhibitory factor enhances lipopolysaccharide-induced fibroblast proliferation by inducing toll-like receptor 4

**DOI:** 10.1186/s12891-016-0895-0

**Published:** 2016-01-26

**Authors:** Zheng-de Xi, Chang-yi Xie, Ye-bin Xi

**Affiliations:** Shanghai Institute of Immunology, Department of Immunology and Microbiology, Shanghai Jiao Tong University School of Medicine, Room 905, Building 5, 280 South Shanghai Chongqing Road, Shanghai, 200025 China

**Keywords:** Macrophage migration inhibitory factor, Toll-like receptor 4, Lipopolysaccharide, L-929 cell line, Connective tissue-derived fibroblast, Cell proliferation, *Escherichia coli*

## Abstract

**Background:**

Fibroblast proliferation is a common manifestation of chronic inflammatory diseases, including rheumatoid arthritis (RA), Crohn’s disease and ulcerative colitis, etc. To alleviate patient suffering, the mechanism underlying fibroblast proliferation should be elucidated.

**Methods:**

CCK-8 assay was used to assess the stimulatory effect of LPS and macrophage migration inhibitory factor (MIF) on fibroblast proliferation. Then, TLR4 expression on fibroblast cell membrane was carried out by confocal scanning microscopy. Finally, real-time fluorescent quantitative PCR and flow cytometry were applied to determine the expression of TLR4 after MIF challenge.

**Results:**

LPS alone directly stimulated the fibroblast proliferation. In addition, MIF showed co-stimulatory effect on LPS-induced fibroblast proliferation. Interestingly, fibroblast overtly expressed TLR4 without stimulation. After MIF stimulation, real-time PCR showed TLR4 mRNA levels were increased by about 33 % in the fibroblasts; in agreement, TLR4 expression on the fibroblast membrane was increased by about 20 %, as shown by flow cytometry.

**Conclusions:**

These findings indicated MIF elevates TLR4 expression in fibroblast, enhancing LPS-induced cell proliferation.

## Background

Rheumatoid arthritis (RA) and Crohn’s disease are common chronic inflammatory diseases. Clinically, RA can cause joint swelling, pain, damage, and even disability; meanwhile, patients suffering from Crohn’s disease usually present with symptoms like constipation, abdominal pain and diarrhea. Both diseases differ in etiology and pathogenic manifestations, but a similar clinical manifestation, with the fibroblast proliferation, playing an important role in both ailments. Therefore, exploring the mechanism underlying fibroblast proliferation is of great significance.

**Fig. 1:**
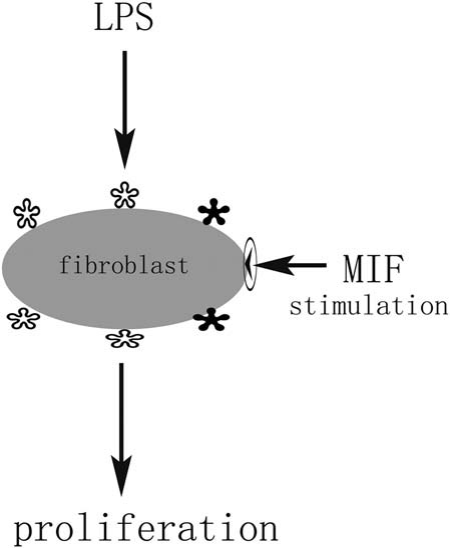
The roles of MIF and LPS in fibroblast proliferation

In a previous study assessing prostaglandin E2 (PGE2) secretion by fibroblasts challenged by lipopolysaccharide (LPS) and macrophage migration inhibitory factor (MIF), we fortuitously found LPS alone stimulates fibroblast proliferation, with MIF playing a co-stimulatory role in enhancing LPS-induced cell proliferation, However, the underlying mechanism is not fully understood. Recent research has reported LPS induces lung fibroblast proliferation through TLR4 signaling[1–4]; in addition, the innate immune response can be regulated by MIF through modulation of Toll-like receptor 4 (TLR4) in macrophage[5]. This study aimed to elucidate the biological function of LPS and MIF in fibroblast proliferation. We propose here that MIF induced TLR4, an LPS receptor, boosting LPS-induced fibroblast proliferation (see Fig. 1). This hypothesis may possibly depict the involvement of MIF in LPS-induced fibroblast proliferation, which leads to synovial hyperplasia (RA) as well as intestinal lumen fibrosis and stenosis (Crohn’s disease).

### Schematic diagram depicting MIF and LPS effects on fibroblast proliferation

In order to unveil the mechanism of the co-stimulatory effect of MIF on LPS-induced fibroblast proliferation, the following hypothesis was proposed (Fig. 1).

 LPS induced fibroblast proliferation via TLR4, and MIF enhanced TLR4 expression. Together, MIF has co-stimulatory function to enhance LPS-induced fibroblast proliferation via Toll-like receptor 4 induction. Blank and solid stars indicated naturally expressed and MIF-induced TLR4, respectively.

## Methods

### Cell line and reagents

The mouse connective tissue-derived fibroblast cell line L-929 was obtained from Shanghai Fu Xiang Biotechnology Co., Ltd. (China). HyClone fetal bovine serum (FBS) was purchased from Shanghai Yu Bo Biological Technology Co., Ltd. (China). LPS (derived from *E. coli* O111:B4), CCK-8 (cell counting kit-8), 4 % paraformaldehyde, sealing medium and trypsin were purchased from Beyotime Institute of Biotechnology Co., Ltd. (China); MIF was obtained from R & D systems (USA); rabbit anti-mouse TLR4 antibody, FITC-conjugated donkey anti-rabbit IgG secondary antibody and donkey serum were purchased from Santa Cruz Biotechnology, Inc. (USA). Quantitative PCR kit was purchased from Wuhan Google Biotechnology Co,. Ltd. (China). PE-conjugated anti-mouse TLR4/MD2 antibody (Miltenyi Biotec, Inc.) was purchased from Shanghai Univ-bio Co., Ltd. (China).

### CCK-8 assay for fibroblast proliferation assessment

L-929 cells were cultured with DMEM containing 10 % FBS, 100 U/ml penicillin and 100 μg/ml streptomycin at 37 °C in a humidified atmosphere with 5 % CO_2_ (Forma Series II CO2 incubator, Thermo Electron Co.). Mouse fibroblast suspension was adjusted to 5 × 10^4^/ml, and 200 μl were seeded per well in 96-well plates. After 1 h incubation (to allow cells to adhere to plates), cells were treated with LPS at various concentrations for 48 h. Then, cell proliferation was assesses by adding 20 μl CCK-8 reagent in each well, followed by incubation at 37 °C for 2 h. Signals were spectrophotometrically measured at 450 nm on a full wavelength microplate analyzer (Molecular Device); data were recorded as optical density (OD). The procedure described above was also used to evaluate the effects of various concentrations of MIF in the presence or absence of 25 μg LPS.

### Immunofluorescence for TLR4 detection

A total of 1 × 10^6^ L-929 cells were seeded on sterile coverslips (0.13 mm thickness) in each 6-well plates, in 1.5 ml of medium. 40 h later, coverslips were gently washed 3 times with PBS and fixed with 4 % paraformaldehyde at room temperature for 20 min. After PBS wash, blocking was carried out with donkey serum (1:10) for 2 h at 37 °C; then, the coverslips were sequentially incubated with rabbit anti-mouse TLR4 antibody (1:50 dilution; 4 °C overnight) and FITC-conjugated donkey anti-rabbit IgG secondary antibody (1:100; 37 °C 1 h). The coverslips were mounted with sealing medium, followed by analysis on a laser confocal scanning microscope (Zeiss LSM-710).

### Real time PCR for TLR4 mRNA expression quantitation

L-929 cells were seeded 5 × 10^5^ per well in 6-well plate treated with MIF at three concentrations, and two replicates per concentration were applied. 48 h later, the medium in each well was aspirated, the attached cells were washed with PBS; after addition of 1 ml Trizopure, total RNA was extracted. RNA purity and concentration were assessed by OD measurement at 260/280 nm on spectrophotometer (Thermo Nanodrop 2000). cDNA was obtained with reverse transcription kit (ReverTra Ace-alpha), and real time PCR was carried out with Toyobo thunderbird SYBR qPCR Mix (quantitative PCR amplification kit). Specific primers were designed and synthesized by Invitrogen Biotechnology (China): TLR4 [NCBI(GenBank):NM_021297], forward, 5'- AGTTTAGAGAATCTCTGGTGGCTGTG-3', and reverse, 5'- TTCCCTGAAAGGCTTGGTCT-3'); β-actin [NCBI(GenBank):NM_007393.3], forward, 5'- CTGAGAGGGAAATCGTGCGT-3', and reverse, 5'-CCACAGGATTCCATACCCAAGA-3'. Thermal cycling conditions included: initial denaturing step at 95 °C for 1 min; 40 cycles of 95 °C for 15 s, 58 °C for 20 s and 72 °C for 20 s; final extension at 72 °C for 5 min. All samples were tested in triplicate on 96-well PCR plate. Relative expression of the TLR4 gene was calculated using the △△Ct method.

### Flow cytometry for detection TLR4 expression on cell membrane

L-929 cells were seeded at a density of 5 × 10^5^ per well in 6-well plate, with MIF added at 375 ng/ml for 48 h. Then, cells were pelleted centrifugation at 300× g for 10 min, and resuspended in 100 μl buffer solution. Afterwards, 10 μl PE-conjugated TLR4-MD2 (CD284) antibody was added for 10 min at 4 °C, and cells were analyzed on flow cytometer (BD Accuri C6).

### Statistical analysis

Statistical analyses were performed with SPSS software (SPSS 19, Inc, Chicago, IL, USA). Data are mean ± SEM, and one-way ANOVA with dunnett's test was used for group comparisons. *P < 0.05 was considered statistically significant.

## Results

### LPS induces mouse fibroblast proliferation

**Fig. 2:**
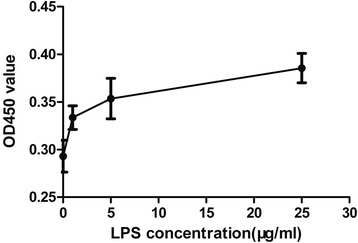
LPS induces proliferation of fibroblast in a dose-dependent manner. Mouse fibroblasts were seeded in 96-well plate. 1 μg, 5 μg, 25 μg/ml LPS were added to various wells; control untreated cells were setup. 48 h later, 20 μl CCK-8 reagent was added into each well for another 2 h. OD450 value was measured to assess cell number. One-way ANOVA with Dunnett's test was used to estimate the differences between the LPS-treated and control groups. The two LPS-treated groups(dot 3 and dot 4)showed more cells with the control group (dot 1). *P < 0.05

After LPS 48 h of incubation, OD450 value were significantly higher in the two LPS-treated groups (5 and 25 μg/ml) compared with control group (P < 0.05), indicating that LPS induced cell proliferation in mouse fibroblasts. Importantly, cell proliferation was induced by LPS in a dose-dependent manner (Fig. 2).

### TLR4 is expressed in mouse fibroblast

**Fig. 3:**
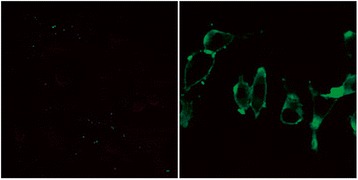
Natural expression of TLR4 in fibroblast. Cells were observed by confocal microscopy. Left image shows the negative control (rabbit anti-mouse TLR4 antibody was not added), with no cell stained by FITC, and cell outline only dimly seen. Right image shows spindle-shaped fibroblasts, with cell membrane staining showing that TLR4 was widely expressed and uniformly distributed on cell membrane. Magnification: ×560

TLR4 expression in the macrophages is downregulated by MIF depletion, leading to the hypo-response of macrophage to LPS[5]. To assess the regulatory role of MIF in fibroblast TLR4 expression, we first examined the expression profile of TLR4 in the mouse fibroblast cell line L-929. Under a confocal microscope, no FITC signal was observed, although clear cell outline was present (Fig. 3, left panel); meanwhile, a spindle-shaped fibroblast outline was high-lighted by FITC signal. The overt staining of cellular membrane suggested that TLR4 was widely expressed and uniformly distributed on the cellular membrane of mouse fibroblasts (Fig. 3).

### MIF enhances TLR4 expression in fibroblast

**Fig. 4:**
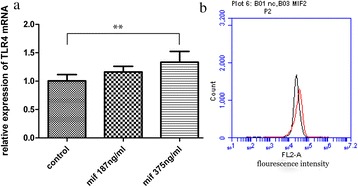
Increased TLR4 expression by fibroblast after MIF stimulation. **a** Mouse fibroblast cells were stimulated with two different concentrations of MIF for 48 h. Transcription levels of the TLR4 gene in mouse fibroblasts were up-regulated by about 33 % after treatment with MIF at 375 ng/ml. **P < 0.01 (**b**) The above finding was further verified by flow cytometry. All cells were stained by PE dye, and 375 ng/ml MIF-treated samples showed clear curve shift to the right of the PE-signal (red peak vs black peak); 20 % elevation of TLR4 in fibroblast cells was observed. Data are representative of 3 independent experiments

Mouse fibroblast cells were stimulated by MIF at 375 ng/ml. Real-time fluorescence quantitative PCR showed that TLR4 mRNA levels were increased by about 33 %. This was further confirmed by flow cytometry, which showed about 20 % increase in TLR4 fluorescence signals after treatment with 375 ng/ml MIF in comparison with control cells (Fig. 4).

### MIF co-stimulates fibroblast proliferation

**Fig. 5:**
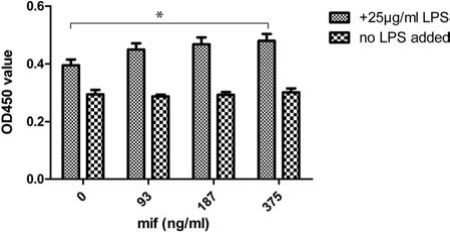
Co-stimulatory effect of MIF on LPS-induced fibroblast proliferation. LPS was used at 25 μg / ml, alongside several MIF concentrations, including 375 ng/ml, 187 ng/ml and 93 ng/ml. In presence of LPS, OD450 value increased with MIF dose, which indicated MIF could co-stimulate fibroblast proliferation. Without LPS, MIF could not stimulate fibroblast proliferation within the dose range tested. This indicated that stimulatory effect of MIF on fibroblast proliferation was indirect, requiring LPS

After treatment with 25 μg/ml LPS, fibroblast proliferation was induced. Meanwhile, MIF could boost LPS-induced fibroblast proliferation. Interestingly, fibroblast cell proliferation increased with MIF dose. These data indicated that LPS alone directly induced fibroblast proliferation, which was further enhanced by MIF. At the concentrations used in these experiments, MIF had no direct stimulating effect on fibroblast proliferation. The stimulatory effect of MIF on fibroblast proliferation was dependent on LPS. These findings strongly support our hypothesis (Fig. 5).

## Discussion

Forty years ago, MIF was firstly proposed by Bloom BR in a study assessing delayed hypersensitivity[6]. It is considered the first cytokine found in human, and is nick-named as IL-0 by some researchers. Because of its capacity to inhibit macrophage migration, it was termed “MIF” (macrophage migration inhibitory factor). It is now known to be widely expressed in various cell types, including activated T cells, macrophages and anterior pituitary gland cells, etc.[7–10]. In addition to macrophage migration inhibition, other functions of MIF have yet to be fully elucidated.

Interestingly, Roger and colleagues reported that MIF-deficient macrophages have reduced TLR4 expression and are hypo-responsive in inducing TNF-α upon LPS and Gram-negative bacteria challenges[5]. These data indicate that MIF is essential for TLR4 expression in macrophages.

Multiple reports have demonstrated that LPS participates in a variety of diseases. For instance, LPS promotes cell proliferation in cultured human small intestinal lamina propria fibroblasts [11], human periodontal ligament fibroblasts [12]and adventitial fibroblasts [13], and each of which might induce corresponding diseases.

Both IL-1R and TLR are homologous in the intracellular adaptor molecule domain known as TIR (Toll-IL-1 receptor) domain[14, 15]. IL-1β induces fibroblast proliferation, as evidenced by many experiments [16–18]. Because of the similarity of the cellular domain, we hypothesized that, combined with its agonist LPS, TLR4 can induce the fibroblast proliferation; indeed, He Z and colleagues reported that LPS induces mouse lung fibroblast proliferation through TLR4 signaling[1]. Our results confirm that LPS stimulates the proliferation of fibroblast L-929, in a dose-dependent fashion (Fig. 2).

Macrophages and fibroblasts can be regarded as basic cells in synovium, with synovial fibroblasts overtly outnumbering macrophages. We hypothesize that fibroblast may also express TLR4, with MIF upregulating TLR4, which contributes to fibroblast proliferation in presence of LPS. As shown above, TLR4 was detected in mouse fibroblast cell line L-929 by laser confocal scanning microscopy. As expected, TLR4 was widely distributed on the cellular membrane (Fig. 3). MIF induced TLR4 expression in fibroblast was also demonstrated by real-time PCR assay, and further confirmed by flow cytometry (Fig. 4). Taken together, MIF, as an internal factor, induces TLR4 expression in fibroblast; this may enhance LPS-induced fibroblast proliferation.

Synovial hyperplasia is an important clinical manifestation of RA, and the underlying mechanism is far to be elucidated. It can be caused by viruses, mycoplasma and chlamydia, but the role of bacterial components has been occasionally reported. Indeed, LPS is an important external factor involved in arthritis occurrence [19–25]. We speculate that LPS may originate from mouth, gut, urogenital tract and respiratory tract in RA patients. Then, bacterial pathogen-produced LPS migrate into the joints through blood circulation, inducing cytokine release and fibroblast proliferation.

## Conclusions

Our data clearly indicate MIF increases TLR4 expression in fibroblast, enhancing the LPS-induced cell proliferation. These findings further describe the roles of LPS and MIF in fibroblast proliferation. Further studies should focus on determining how MIF induces TLR4, exploring the related signal transduction pathway. Blockage of LPS-TLR4 and MIF-CD74 signaling may be promising in treating synovial hyperplasia in RA.

## Availability of supporting data

None.

## Requesting deposition of data

None.

## Abbreviations

ANOVA: Analysis of variance
CCK-8: cell counting kit-8
DMEM: Dulbecco’s Modified Eagle Medium
LPS: lipopolysaccharide
MIF: macrophage migration inhibitory factor
qPCR: quantitative polymerase chain reaction
RA: rheumatoid arthritis
TIR: Toll-IL-1 receptor
TLR4: Toll-like receptor 4
